# Novel ultrasound features and diagnostic clues of gastric-type endocervical adenocarcinoma: a case series

**DOI:** 10.3389/fonc.2025.1572438

**Published:** 2025-05-14

**Authors:** Liwen Yang, Yangyang Wang, Jian Cai, Ying Xiong, Juan Li, Qi Zhou, Nan Ye, Hua Lai, Tianjiao Liu, Liuying Zhou

**Affiliations:** ^1^ Department of Ultrasonography, Chengdu Women’s and Children’s Central Hospital, School of Medicine, University of Electronic Science and Technology, Chengdu, China; ^2^ Department of Gynecology and Obstetrics, Chengdu Xinjin District Maternal and Child Health Care Hospital, Chengdu, China; ^3^ Department of Pathology, Chengdu Women’s and Children’s Central Hospital, School of Medicine, University of Electronic Science and Technology, Chengdu, China; ^4^ Department of Gynecology, Chengdu Women’s and Children’s Central Hospital, School of Medicine, University of Electronic Science and Technology, Chengdu, China; ^5^ Department of Diagnosis and Treatment for Cervical Disease, Chengdu Women’s and Children’s Central Hospital, School of Medicine, University of Electronic Science and Technology, Chengdu, China; ^6^ Department of Radiology, Chengdu Women’s and Children’s Central Hospital, School of Medicine, University of Electronic Science and Technology, Chengdu, China

**Keywords:** gastric-type endocervical adenocarcinoma, ultrasonic diagnosis, preoperative diagnosis, synchronous mucinous metaplasia and neoplasia of the female genital tract, cosmos sign, endometrial cavity fluid

## Abstract

**Background:**

Gastric-type endocervical adenocarcinoma (G-EAC) is a rare and aggressive subtype of cervical cancer which is not associated with human papillomavirus (HPV) infection but has poor prognosis because of its high invasiveness and resistance to chemoradiotherapy. The early and accurate diagnosis of G-EAC is challenging owing to its nonspecific symptoms and relatively normal cytological and histological manifestations.

**Methods:**

The present study retrospectively analyzed the demographic and clinical characteristics, and cervical medical imaging features of 10 patients diagnosed with G-EAC at our institution during a five-year period. Postoperative cervical pathological features were examined, and followed-up information was collected. Novel ultrasonographic features of G-EAC were also summarized.

**Results:**

The patients aged 24–70 years (mean: 49.6 ± 11.6). Their clinical presentations included vaginal discharge (60%), irregular vaginal bleeding (40%), and contact bleeding (30%). Nine patients were HPV negative. Ultrasound examination revealed that there were two, three, two, and two cases of types I (multicystic), II (cystic-solid), III (solid), and IV (nearly normal cervix) G-EAC, respectively. There were four CA199 + and two CA125 + cases. Pathology examination confirmed two cases of synchronous mucinous metaplasia and neoplasia of the female genital tract and one case of Peutz–Jeghers syndrome and multiple gastrointestinal polyps. Ultrasonography for cystic lesions revealed a “cosmos sign”. Two patients with types I and II G-EAC exhibited vesicular echoes involving the lower uterine segment. In four cases, vesicular echoes were observed within the myometrium. This case series highlights the heterogeneous manifestations, complex imaging patterns, and multifaceted pathology of G-EACs.

**Conclusions:**

Ultrasonography can facilitate the early diagnosis of G-EAC for relatively specific features, such as “cosmos signs” and “vesicular implantation signs.” The latter refers to ultrasound manifestations of multicystic or cystic-solid lesions of the cervix accompanied by vesicular lesion in the lower uterine segment and/or vesicular implantation in the myometrium.

## 1 Introduction

Gastric-type endocervical adenocarcinoma (G-EAC) is a rare and aggressive subtype of cervical mucinous adenocarcinoma, accounting for 10–15% of all cervical adenocarcinomas (1, 2). Unlike other types of cervical cancers, G-EAC is the most common non-human papillomavirus (HPV)-associated cervical cancer (3). It is characterized by gastric (pyloric gland-like) metaplasia; however, its exact pathogenesis remains unclear.

Previous studies have suggested that G-EAC may be associated with cervical precursor lesions and Peutz–Jeghers syndrome (PJS), with approximately 10% of patients with G-EAC diagnosed with PJS (4). G-EAC often presents with nonspecific symptoms, such as persistent watery vaginal discharge, abnormal vaginal bleeding, abdominal pain, and bloating, sometimes with a pelvic mass as the initial symptom (5). Because of its highly invasive nature, G-EAC easily metastasizes via blood and lymphatic vessels and is relatively insensitive to radiotherapy and chemotherapy, leading to poor prognosis and low five-year survival rates (6).

The pathological characteristics of G-EAC include well-differentiated glands with minimal heterogeneity, making cytological screening and routine cervical biopsies challenging and prone to misdiagnosis. Currently, repeated multiple-site cervical biopsies, ultrasound-guided cervical puncture biopsies, and cervical conization can improve diagnostic accuracy to approximately 50% (7). Provided the insidious nature of G-EAC and its poor prognosis, preoperative medical imaging methods, particularly those with higher accuracies, are important in facilitating early diagnosis, guiding biopsy sampling, and enhancing patients’ prognosis.

Presently, medical imaging studies on G-EAC have primarily focused on its magnetic resonance imaging (MRI) features, with fewer studies summarizing its ultrasound characteristics (8–11). This study retrospectively analyzed the clinical data and ultrasound features of 10 patients with G-EAC diagnosed by histopathology at our institution in past five years and reported several novel ultrasound features of G-EAC, such as the “vesicular implantation sign” and “cosmos sign,” to increase awareness among clinicians and sonographers about G-EAC and avoid misdiagnosis and missed diagnosis.

## 2 Materials and methods

### 2.1 Case selections and study design

We retrospectively analyzed the clinical data and ultrasound features of patients with G-EAC confirmed on postoperative pathological examinations at Chengdu Women’s and Children’s Central Hospital between August 2018 and April 2023. All patients underwent preoperative ultrasound examination. The inclusion criterion was pathologically confirmed G-EAC; the staging of which was performed according to the International Federation of Gynecology and Obstetrics (7, 12, 13). The staging of G-EAC is generally categorized into four main stages (I-IV). In stage I, carcinoma is confined to the cervix and extension to the uterine corpus is disregarded. In stage II, the tumor invades beyond the uterus but does not involve the lower third of the vagina or the pelvic wall. In stage III, the tumor involves the lower third of the vagina and/or pelvic wall and pelvic and/or para-aortic lymph nodes, causes kidney hydronephrosis or destroys kidney function, or presents with a combination of these features; In stage IV, the carcinoma extends to adjacent pelvic organs such as the bladder and rectum, or presents with distant metastases involving extrapelvic sites. The exclusion criteria included concurrence of other malignant tumors, history of prior radiotherapy or chemotherapy, and history of pelvic surgery. The following sociodemographic and clinical data were collected; age, clinical symptoms including vaginal discharge, abnormal vaginal bleeding, tumor markers (CA125, CA199, CEA, and SCC), medical imaging findings including pelvic ultrasound and MRI, HPV test results, cervical cytology screening results, cervical biopsy findings, pathological findings, and the presence of PJS. Ten patients were also followed up postoperatively until the present, were lost to follow-up, or died. During the follow-up period, detailed information was recorded, including patient follow-up status, detection of malignant tumors in other organs, administration of chemoradiotherapy, occurrence of metastasis or other complications, physical examination findings, and self-reported health status.

### 2.2 Ultrasonography

This study applied instruments similar to those described in our previous study, which included Samsung WS80a, GE Voluson E8, and Mindray R9 color Doppler ultrasound systems (14). The vaginal probe frequency was set at 5–9 MHz, and the abdominal probe frequency was 3.5 MHz. Patients were mostly required to empty their bladders and rectum before the examination. Depending on the patient’s sexual history, transvaginal or transrectal ultrasonography was performed. For cases in which the intracavitary ultrasound was suboptimal because of a large cervical or pelvic mass, the patient was instructed to fill the bladder for transabdominal ultrasound examination. The uterus and adnexa were routinely scanned, focusing on the following: 1. cervical size, shape, echotexture, and presence of a mass; 2. mass characteristics (cystic, solid, or cystic-solid mixed), specific location, size, margins, internal echogenicity, and blood flow signals; 3. extent of tumor invasion, including involvement of the lower uterine segment, myometrium, parametrium, vagina, bladder, and rectum; 4. presence of intrauterine fluid accumulation; 5. presence of enlarged pelvic lymph nodes, ascites, or bilateral ovarian abnormalities. Three ultrasonographers performed gynecological ultrasound examinations on patients with suspected cervical tumors. In this study, we adopted a definition of the “cosmos sign” consistent with previous reports. The “cosmos sign” refers to the honeycomb-like clustering of small cysts surrounded by larger cystic structures. This feature may represent a relatively distinctive manifestation of G-EAC, aiding its differentiation from other types of cervical cancer (15, 16). Additionally, “vesicular implantation sign” was defined as multicystic or cystic-solid lesions of the cervix accompanied by vesicular lesion in the lower uterine segment and/or vesicular implantation in the myometrium (14).

### 2.3 Statistical analysis

SPSS software (version 26.0) was used for the statistical analysis. Quantitative data are expressed as mean ± standard deviation, and categorical data are presented as percentages (%). The assessment of observer agreement included all three sonographers and was conducted using 10 cases of G-EAC and 90 cases of other types of cervical cancer. Interobserver reliability for cervical ultrasound diagnosis was determined using Fleiss’ kappa. Intraobserver consistency was assessed via Cohen’s kappa (κ). The κ values and their corresponding 95% confidence intervals were computed with SPSS software and interpreted according to the criteria established by Landis and Koch. κ values greater than 0 up to 0.2 signify slight agreement, values greater than 0.2 up to 0.4 signify fair agreement, values greater than 0.4 up to 0.6 signify moderate agreement, values greater than 0.6 up to 0.8 signify substantial agreement, and values greater than 0.8 up to 1.0 signify almost perfect agreement (17).

## 3 Results

### 3.1 Demographic and clinical features of patients

This study included 10 patients with G-EAC aged 24–70 years (mean age; 49.6 ± 11.6 years) (**Table 1**). Seven (70%) patients were postmenopausal. Vaginal discharge was observed in six cases (60%), irregular vaginal bleeding in four (40%), contact bleeding in three (30%), abdominal pain or bloating in one (10%), and an abdominal mass in one (10%). Gynecological examination revealed one case (10%) with a significantly enlarged, firm cervix and six cases (60%) with moderately enlarged cervices. Two patients (20%) had nodules and one patient displayed a cauliflower-like cervix. The auxiliary examinations indicated that nine cases (90%) were HPV-negative, and one case (10%) was HPV-positive. The ThinPrep Cytologic test shows atypical squamous cells of undetermined significance (ASC-US) in two cases (20%), atypical glandular cells in two (20%), and high-grade squamous intraepithelial lesion in one (10%). Cervical biopsy found cervical adenocarcinoma in two cases (20%) and low-grade squamous intraepithelial lesion in two (20%). Preoperative tumor marker evaluation showed elevated CA199 in four cases (40%) and elevated CA125 in two (20%).

**Table 1:** Clinical characteristics of patients with G-EAC.

Case	Age	Clinical Symptoms	Cervical Appearance	Tumor Markers	HPV	TCT	Cervical Biopsy	G-EAC Stage^a^	Invasion/Metastasis	Metaplasia	Concurrence of PJS	MRI/CT Findings
1	54	Vaginal discharge	Slightly enlarged, smooth, firm	Negative	66, 68 (+)	ASC-US	/	II	/	/	/	Multicystic lesion in the cervical region
2	49	Lower abdominal discomfort, vaginal bleeding on contact	Enlarged, smooth, firm	CA199 202.16 U/ml	Negative	/	Adenocarcinoma	IV	Parametrial and vaginal invasion, metastasis to bilateral ovaries and pelvic lymph nodes	/	/	Suspected cervical cancer; unclear upper vaginal and parametrial structures, thickened left adnexa, visible lymph nodes
3	46	Vaginal bleeding on contact, slight vaginal discharge	Thickened, with a 3.5 cm × 2.0 cm × 2.0 cm cauliflower-like growth at cervical os	/	Negative	HSIL	Adenocarcinoma at 3/6/9/12 positions	I	/	/	/	Cervical adenocarcinoma involving vagina and rectum
4	49	Recurrent vaginal discharge	Moderately enlarged, smooth, with a 3 cm × 4 cm bulge palpable at the isthmus	CA199 292.7 U/ml	Negative	NILM	LSIL	IV	Unilateral ovarian metastasis	/	/	Suspected endometrial and cervical glandular cysts
5	47	Intermittent vaginal bleeding	Moderately enlarged, eroded, firm	CA125 81.2 U/ml	Negative	ASC-US	/	II	Vaginal vault invasion	/	/	Adenomyosis with intrauterine hematometra
6	51	Pelvic mass	Slightly enlarged, smooth, mildly eroded	CA125 46.4 U/ml; CA199 53.51 U/ml	Negative	AGC	LSIL	II	/	Synchronous mucinous borderline tumor of left ovary	/	Cervical Nabothian cysts, suspected mucinous cystadenoma of left adnexa
7	43	Prolonged menstruation, irregular spotting, vaginal bleeding on contact	Smooth, medium-sized	/	Negative	NILM	/	In situ adenocarcinoma	/	/	/	Enlarged cervix
8	70	Vaginal discharge for several years, postmenopausal bleeding	Atrophic	CA199 > 700.00 U/ml	Negative	NILM	Unsatisfactory colposcopy	I	/	Synchronous high-grade mucinous endometrial adenocarcinoma	/	Cervical Nabothian cysts
9	24	No history of sexual activity, irregular menstruation, spotting with vaginal discharge	Not examined	/	Negative	/	aLEGH	/	/		✓	Suspected gastric-type mucinous adenocarcinoma of the cervix
10	63	Vaginal discharge for over 2 years	Smooth, atrophic	/	Negative	AGC	/	III	Parametrial invasion, metastasis to right pelvic lymph nodes	/	/	Multiple cystic lesions in upper cervical segment and posterior uterine wall, suspected neoplastic changes

a based on the International Federation of Gynecology and Obstetrics (FIGO) grading criteria of G-EAC.

aLEGH, Atypical Lobular Endocervical Glandular Hyperplasia; ASC-US, Atypical Squamous Cells of Undetermined Significance; CA125, Cancer Antigen 125; CA199, Cancer Antigen 199; CEA, Carcinoembryonic Antigen; CT, computed tomography; FIGO, International Federation of Gynecology and Obstetrics; G-EAC, Gastric-type Endocervical Adenocarcinoma; gAIS, Gastric-type Adenocarcinoma in Situ; HPV, Human Papillomavirus; HSIL, High-grade Squamous Intraepithelial Lesion; LSIL, Low-grade Squamous Intraepithelial Lesion; MRI, Magnetic Resonance Imaging; PJS, Peutz-Jeghers Syndrome; TCT, ThinPrep Cytologic Test.

### 3.2 Postoperative pathological findings

**Figure 1:**
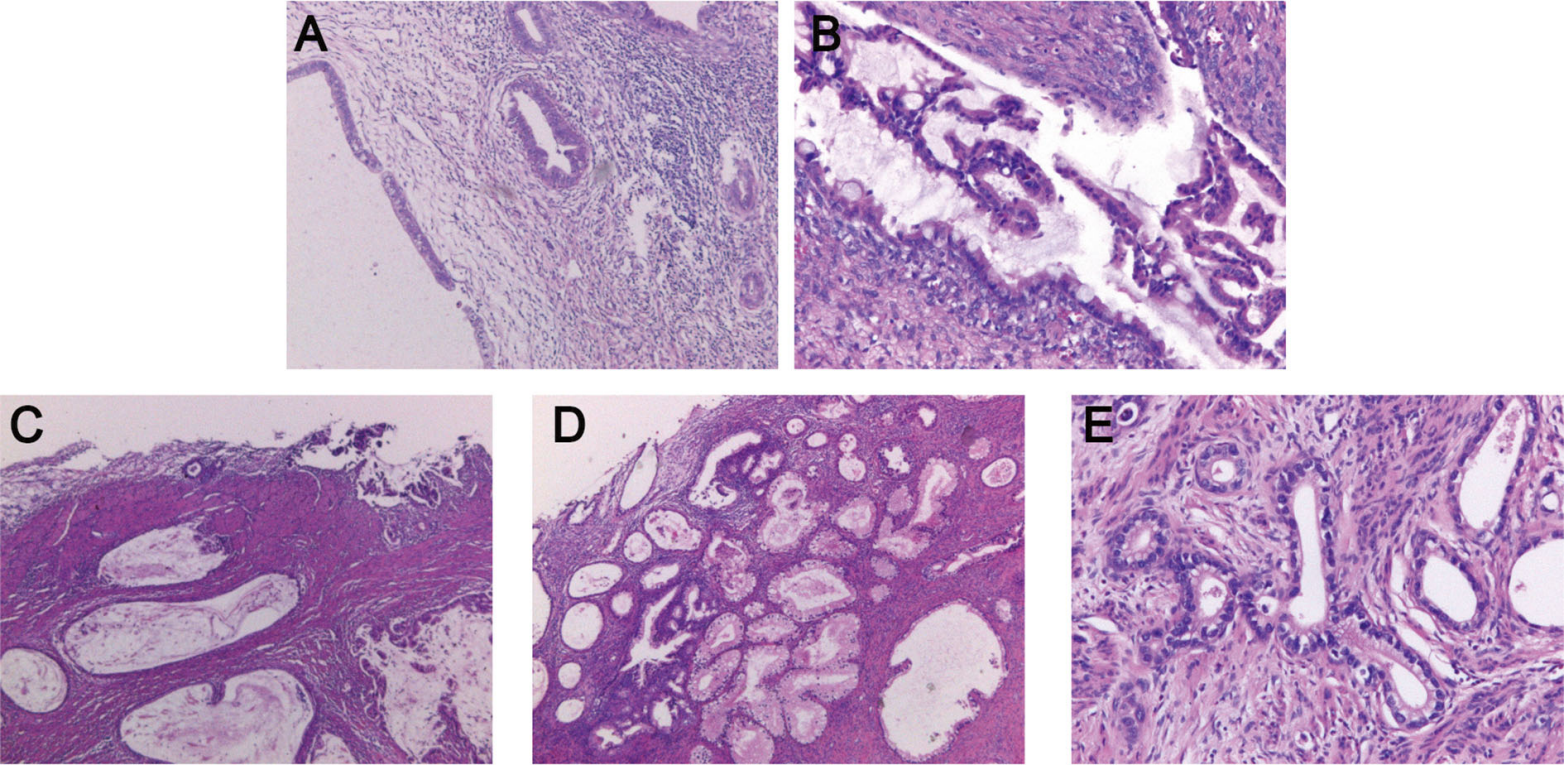
Postoperative pathological examination of G-EAC. **(A)** H&E staining of the lower uterine segment in case 6 shows the vesicular lesions involving the lower segment of the uterine cavity. **(B)** H&E staining of left adnexa in case 6 reveals a mucinous borderline tumor. **(C)** H&E staining of endometrium in case 8 demonstrates highly differentiated endometrioid mucinous adenocarcinoma. **(D)** H&E staining of the cervix in case 10 reveals gastric-type adenocarcinoma. **(E)** H&E staining of the uterus in case 10 shows cystic lesions infiltrating the myometrium. H&E, Hematoxylin and Eosin; G-EAC, gastric-type endocervical adenocarcinoma.

**Figure 2:**
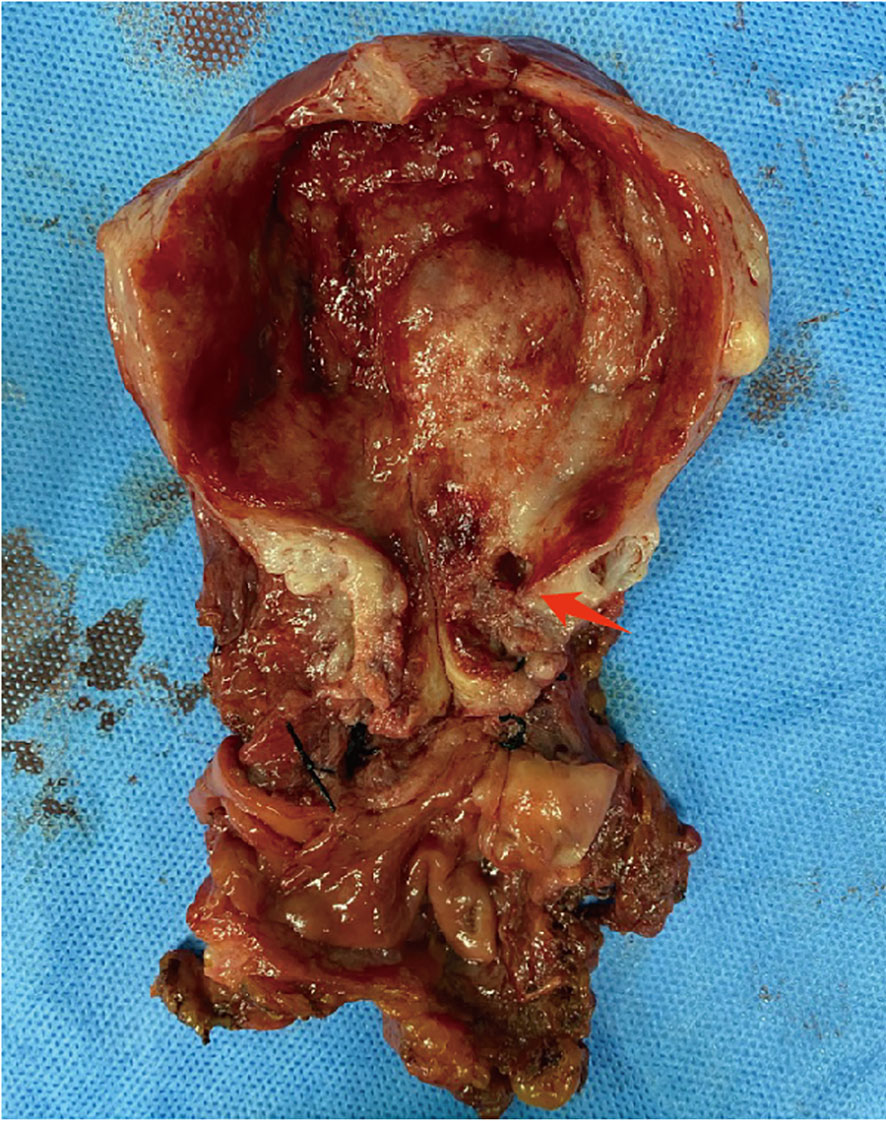
Macroscopic view of resected uterine and cervix in case 10. The cystic echo within the uterine myometrium (red arrow).

The postoperative pathological findings of the patients are described in **Table 1** and presented in **Figure 1**. **Table 2** data demonstrate the substantial interobserver and intraobserver agreements for cervical ultrasound diagnosis, with a Fleiss’ kappa (interobserver) of 0.719 and Cohen’s kappa (intraobserver) of 0.737. There were two (20%), three (30%), one (10%), and two (20%) cases of stages I, II, III, and IV G-EAC, respectively. Two patients had parametrium invasion, two had vaginal fornix invasion, two had ovarian metastases, and two had pelvic lymph node metastases. Hematoxylin and eosin (H&E) staining of the lower uterine segment in case 6 showed vesicular lesions involving the lower segment of the uterine cavity (**Figure 1A**). Two patients presented with synchronous mucinous metaplasia and neoplasia of the female genital tract (SMMN-FGT); one with a mucinous borderline tumor of the left ovary (**Figure 1B**) and one with highly differentiated mucinous adenocarcinoma of the endometrium (**Figure 1C**). Additionally, one case of gastric-type adenocarcinoma *in situ* (gAIS, **Figure 1D**) and one case of atypical lobular endocervical glandular hyperplasia (aLEGH) were diagnosed. H&E staining and a macroscopic view of the resected uterus and cervix in case 10 indicated cystic lesions infiltrating the myometrium (**Figures 1E**, **2**). Additionally, one case involved a 24-year-old woman with PJS, characterized by multiple polyps in the stomach and duodenum and a family history of the condition (the patient’s father and brother had scattered black spots on the lips, oral mucosa, fingertips, and toe tips).

**Table 2:** Assessment of inter- and intraobserver reliability for cervical ultrasound diagnosis.

Agreement	κ	95% CI	Level of agreement
Interobserver Agreement^a^	0.719	0.606-0.862	Substantial
Intraobserver Agreement^b^	0.737	0.617-0.879	Substantial

a Fleiss’ kappa.

b Cohen’s kappa.

CI, Confidence Interval.

### 3.3 Medical imaging findings

#### 3.3.1 MRI and computed tomography findings

**Figure 3:**
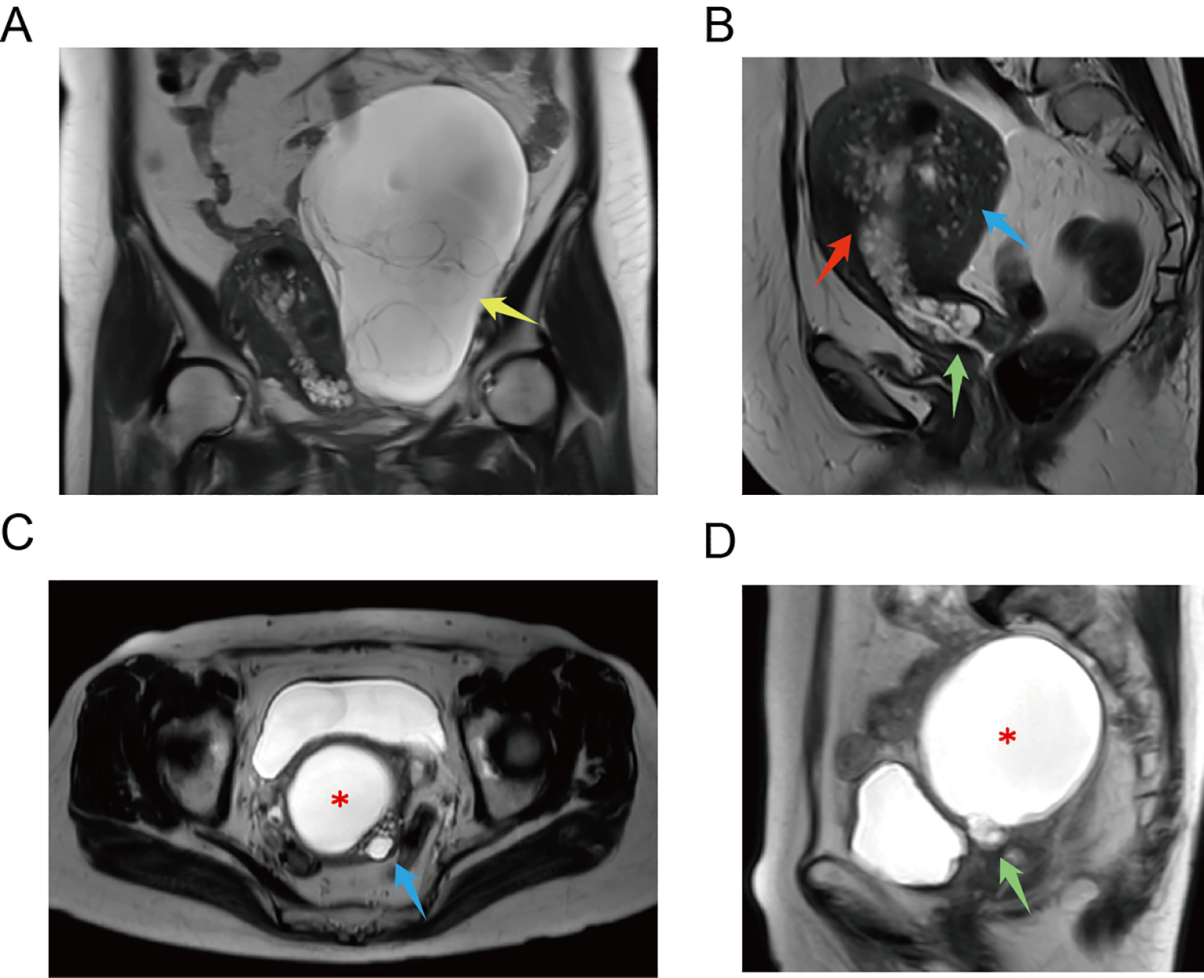
MRI examination of G-EAC. **(A)** Coronal T2-weighted image of pelvic cavity in case 6 demonstrates a multilocular cystic mass in the left adnexal region. Postoperative histopathological examination confirms that it was a SMMN-FGT. Multilocular cystic mass (yellow arrow). **(B)** Sagittal T2-weighted image of pelvic cavity in case 6 demonstrates multiple cystic signals extending from the cervix to the lower uterine cavity. Multiple small cystic signals are observed infiltrating the myometrium. Multilocular cystic lesion in the lower uterine cavity (red arrow), multiple cystic lesions in the myometrium (blue arrow), and a cystic lesion in the cervix (green arrow). **(C)** Transverse T2-weighted image of pelvic cavity in case 10. ECF (asterisk) and multiple cystic lesions of various sizes in the uterine myometrium (blue arrow). **(D)** Sagittal T2-weighted image of pelvic cavity in case 10. ECF (asterisk) and multiple cystic lesions in the uterine isthmus (green arrow). ECF, endometrial cavity fluid; G-EAC, gastric-type endocervical adenocarcinoma; SMMN-FGT, synchronous mucinous metaplasia and neoplasia of the female genital tract.

The MRI and CT findings are listed in the final column of **Table 1**. Preoperative MRI/CT examination revealed suspected cervical cancer in four cases, cervical Nabothian cysts in four, cervical enlargement in one, and no significant abnormalities in one. Additionally, we report two cases of vaginal invasion, one case of parametrial invasion, one case of ovarian metastasis, one case of lymph node metastasis, and one case of rectal metastasis. Representative MRI findings are shown in **Figure 3**. Coronal (**Figure 3A**) T2-weighted images of the pelvic cavity in case 6 revealed a multicystic mass located in the left adnexal region. Postoperative histopathological examination confirmed the diagnosis of SMMN-FGT. The sagittal (**Figure 3B**) T2-weighted images of the pelvic cavity in case 6 demonstrated multiple small cystic signals infiltrating the myometrium. Additionally, the transverse (**Figure 3C**) and sagittal (**Figure 3D**) T2-weighted images of the pelvic cavity in case 10 demonstrated multiple cystic lesions of varying sizes within the uterine myometrium and uterine isthmus.

#### 3.3.2 Ultrasonography findings

**Figure 4:**
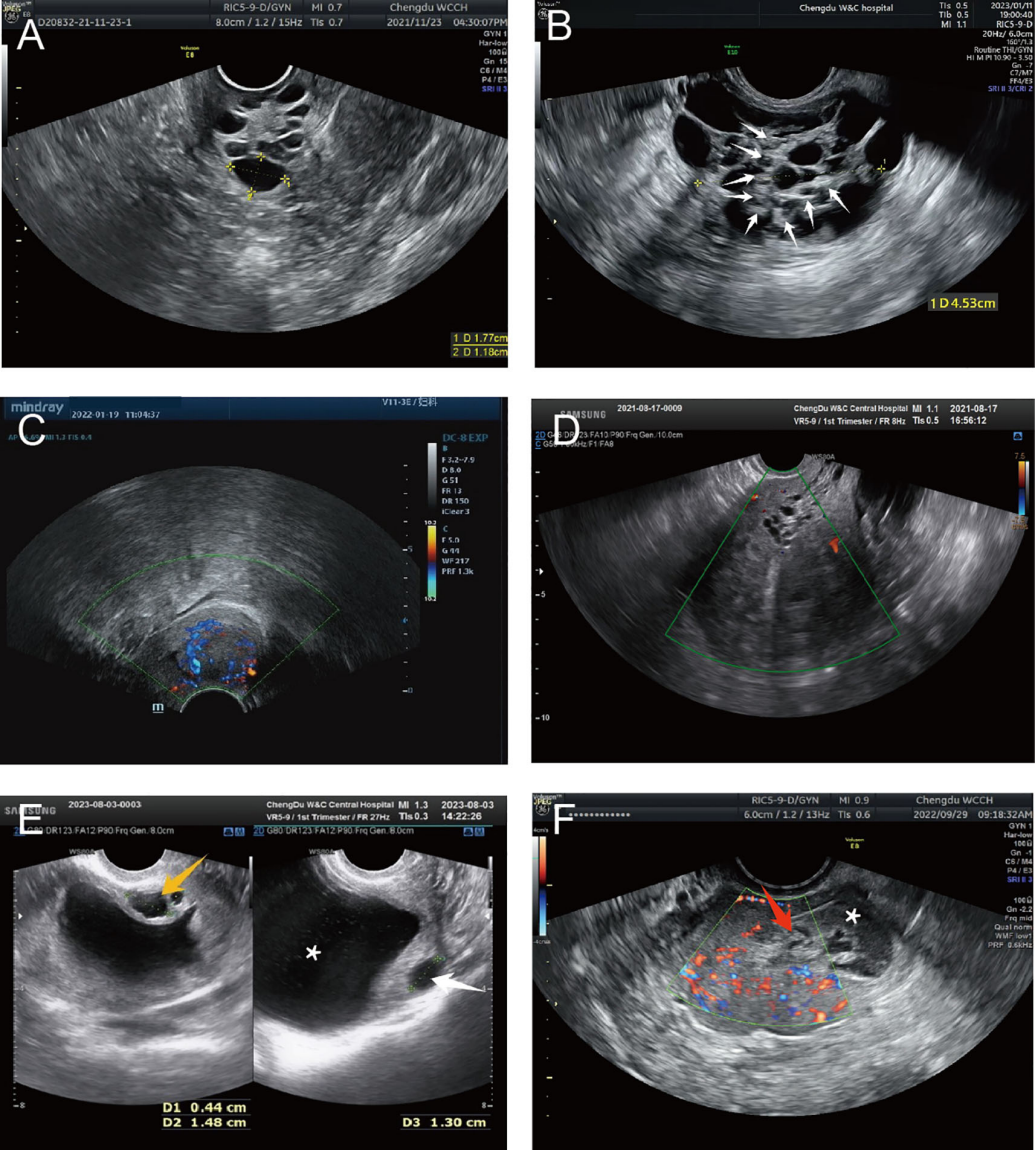
Representative ultrasound images of G-EAC. **(A)** Ultrasound image of type I G-EAC shows the multilocular cystic lesion in the cervix. **(B)** Ultrasound image of type II G-EAC demonstrates a multilocular cystic-solid mass, with smaller cysts surrounded by larger cysts, presenting a “cosmos sign.” Solid echoes (white arrows). **(C)** Type III G-EAC appears as a solid hypoechoic mass with poorly defined borders and no apparent capsule. CDFI exhibits abundant blood flow signals. **(D)** Cervical and uterine ultrasound images of case 6 shows vesicular echoes extending from the cervix to the lower uterine segment. **(E)** Cervical and uterine ultrasound images of case 10 demonstrates ECF and multiple cervical cystic echoes. Vesicular implants are observed within the myometrium. Multilocular cystic cavities in the cervix (orange arrows), intramural vesicular implants (white arrows), and ECF (asterisk). **(F)** Uterine CDFI of case 8 (axial plane). A slightly hyperechoic area is noted in the right uterine cornu with indistinct boundaries from the myometrium. Abundant punctate and linear blood flow signals are detected. Postoperative pathology confirms SMMN-FGT. Cornua uteri (red arrows) and ECF (asterisk). CDFI, color Doppler flow imaging; ECF, endometrial cavity fluid; G-EAC, gastric-type endocervical adenocarcinoma; SMMN-FGT, synchronous mucinous metaplasia and neoplasia of the female genital tract.

Detailed ultrasonography results are listed in **Table 3**. Cervical masses were observed in eight cases (80%) with varying degrees of cervical enlargement. Based on ultrasound characteristics, the cervical lesions in G-EAC were categorized into four types; I, multicystic (two cases, 20%), characterized by a multicystic structure, with some lesions occupying the entire cervix (**Figure 4A**), II, multiple cystic-solid (three cases, 30%), presenting a mixed cystic-solid structure with low isoechoic solid components that lacked clear boundaries and capsules, accounting for < 80% of the lesion volume, sometimes appearing nestled between cystic compartments (**Figure 4B**), III, solid (two cases, 20%), comprising solid lesions with low isoechoic internal echoes, poorly defined boundaries, and no evident capsules, in which the solid component comprised > 80% of the lesion, with a maximum diameter of 3.0 cm (**Figure 4C**), and IV, nearly normal cervix (two cases, 20%), in which the cervix appeared nearly normal in size and shape, with sparse small cystic echoes scattered throughout. The cystic components in types I and II shared similar features, with diameters ranging from 0.2–3.0 cm. In some cases, small cysts appeared honeycomb-like, with larger cysts surrounding smaller ones, resembling the “cosmos sign” observed in MRI (**Figure 4B**). Notably, one patient displayed features of types I and III, with a multicystic structure in the upper cervix and solid protrusion at the external os.

**Table 3:** Ultrasound characteristics of 10 patients.

Case	Appearance and Ultrasound Classification of Cervical Lesion	Lesion Location	Lesion Size(cm)	Involvement of Lower Uterine Cavity	Involvement of Myometrium	Pathological findings	Intrauterine Fluid	Adnexal Mass	Blood Supply to Cervical Lesion	Ascites	Parametrial Invasion/Vaginal Involvement
1	Type II + "cosmos" sign	Diffuse	5.2 × 3.1 × 4.4	/	Scattered cysts in myometrium, largest 0.6 × 0.8 cm in uterine fundus	Myometrial infiltration	/	/	/	/	/
2	Type III	Lower segment	3.9 × 2.6	/	/	/	Small amount	/	Rich	/	Parametrial
3	Type III	Lower segment	2.5 × 2.2 × 2.7	/	/	/	/	/	Rich	/	/
4	Type II + "cosmos" sign	Middle-upper segment	4.0	Multiple small cystic echoes (<0.5 cm)	/	Involvement of lower uterine segment	/	/	Moderate	/	/
5	Type IV	/	/	/	Scattered cysts of 0.3–0.5 cm diameter in myometrium	Deep myometrial infiltration	Moderate intrauterine fluid before external curettage	/	/	/	/
6	Type I	Upper segment	/	Multiple cystic echoes <0.5 cm	Scattered cysts 0.2–0.4 cm in myometrium	Involvement of lower uterine segment, myometrial infiltration	/	Left ovarian mucinous borderline tumor (intestinal type), benign Brenner tumor	Small amount	/	/
7	Type I + III	Upper and lower segments	Cystic mass(3.0), solid vegetation *(*3.0)	/	/	/	/	/	Moderate	/	/
8	Type IV	/	/	/	/	/	Moderate intrauterine fluid	/	Small amount	/	/
9	Type II + "cosmos" sign	Diffuse	5.6 × 4.7 × 4.6	/	/	/	/	Left ovarian serous cystadenoma	Rich	/	/
10	Type I	Upper segment	2.0	/	Multiple cysts in myometrium, largest one (1.0 × 1.4 cm) in left posterior wall	Deep myometrial infiltration	Large amount	/	Small amount	/	/

Ultrasonography can depict the location of the G-EAC and its invasion into adjacent organs. In two patients with types I and II disease, vesicular echoes were detected involving the lower uterine segment, and postoperative pathology confirmed lower uterine segment involvement (**Figures 3B**, **4D**). In four cases, vesicular echoes were observed within the myometrium, and postoperative pathology confirmed myometrial invasion (**Figures 4E**, **4F**). Among the 10 patients, three had lesions located in the upper cervical region, two in the lower cervical region, and two in the entire cervix. One patient presented with a cystic mass in the upper cervical region. A solid neoplasm was observed at the external cervical os, with its pedicle positioned at the 6 o’clock position in the midcervical region.

Moreover, ultrasonography reported endometrial cavity fluid (ECF) in four cases (40%) and adnexal masses in two cases, one of which involved a unilocular cystic mass that was pathologically diagnosed as serous cystadenoma and the other involved a multicystic mass that was pathologically diagnosed as borderline ovarian mucinous cystadenoma (intestinal type) accompanied by a benign Brenner tumor. Preoperative ultrasound suggested paracervical infiltration in one patient (10%) and no ascites in any patient. Color Doppler ultrasound revealed abundant blood supply in solid lesions, whereas multicystic or cystic-solid lesions exhibited low or moderate blood supply. Spectral Doppler imaging revealed a moderate vascular resistance.

### 3.4 Postoperative follow-up outcomes

**Table 4** summarizes the follow-up outcomes of ten patients who underwent surgery for G-EAC. Among them, seven patients remain under regular follow-up, two were lost to follow-up, and one died three years ago. One patient developed a postoperative secondary malignancy (gastric cancer), and three patients underwent chemoradiotherapy, with one completing eight cycles and the other completing 28 cycles. To date, no cases of metastases have been reported. Complications have been minimal, with only one case of hydronephrosis requiring ureteral stent placement and one case of newly diagnosed pulmonary tuberculosis unrelated to gynecologic pathology.

**Table 4:** Postoperative follow-up outcomes of 10 patients.

Case	Follow-up Status	Date of Surgery	Malignant tumor found in other organs	Chemoradiotherapy	Metastasis	Other Complications	Notes
1	Ongoing follow-up	Jan-2022	None	None	None	None	—
2	Lost to follow-up	Oct-2021	—	—	—	—	—
3	Ongoing follow-up	Jan-2022	—	Completed all scheduled chemoradiotherapy	—	—	No subjective symptoms reported by the patient.
4	Deceased (3 years ago)	Dec-2018	—	—	—	—	—
5	Lost to follow-up	Nov-2021	—	—	—	—	—
6	Ongoing follow-up	Aug-2021	None	Completed all scheduled chemoradiotherapy	None	None	No abnormalities found during follow-up.
7	Ongoing follow-up	Dec-2022	Gastric cancer	8 cycles	None	None	—
8	Ongoing follow-up	Sep-2022	None	None	None	None	No postoperative follow-up examinations were conducted; the patient reported no subjective symptoms.
9	Ongoing follow-up	Feb-2023	None	None	None	None	Recently diagnosed with pulmonary tuberculosis; gynecologic examination showed no abnormalities.
10	Ongoing follow-up	Aug-2023	None	28 cycles	None	Hydronephrosis	Underwent ureteral stent placement to relieve obstructive hydronephrosis

## 4 Discussion

G-EAC is a rare and aggressive subtype of cervical adenocarcinoma associated with a poor prognosis, which requires early diagnosis. This study retrospectively analyzed the clinical features and ultrasound findings of 10 patients with G-EAC to enhance awareness and provide diagnostic insights.

The patients’ mean age was 49.6 years, predominantly postmenopausal women, aligning with previous reports (18). Their clinical presentations included vaginal discharge, irregular vaginal bleeding, contact bleeding, pelvic masses, and non-specific symptoms. Interestingly, 90% of the patients were HPV-negative, indicating that G-EAC may not be associated with HPV infection, unlike cervical squamous cell carcinoma (19). Therefore, G-EAC should be considered in postmenopausal women presenting with vaginal discharge or irregular bleeding, although HPV testing is negative.

Previous studies have shown that some patients with G-EAC initially present with abdominal pain, distension, or pelvic masses. This may be explained by two factors. First, the highly invasive nature of G-EAC leads to distant metastases even in the early stages, with ovarian metastases being the most common (15, 20). Second, G-EAC may be associated with SMMN-FGT, in which gastric-type mucinous lesions spread through the reproductive tract, involving the endometrium, fallopian tubes, and ovaries (18). Here, case 6 exemplifies the latter, presenting with a large multicystic lesion in the left ovary, the postoperative pathology of which confirmed it as SMMN-FGT. Therefore, multifocal gastric-type mucinous lesions should be suspected if gastric-type mucinous cervical lesions are detected. Differentiating ovarian metastases from G-EAC and SMMN-FGT remains challenging (21).

Ultrasonography aids in evaluating cervical lesions. Here, G-EAC cases were categorized into four types based on sonographic features; multicystic, cystic-solid, solid, and normal cervix. Multilocular cystic and cystic-solid types accounted for 50% of cases, consistent with the origin of G-EAC from endocervical glands (22). We found that although ultrasonography demonstrated high sensitivity for detecting gastric-type cervical lesions, its specificity was limited, which is consistent with previous publications. Types I and II lesions are difficult to distinguish from benign glandular lesions (9, 11, 23–27). This study identified novel diagnostic clues that may enhance the specificity of G-EAC detection. In two cases (cases 4 and 6) classified as types I and II, cystic echoes extended from the upper cervix to the lower uterine segment. Dense cystic echoes (<0.6 cm) with no significant blood flow signals were observed, and postoperative pathology confirmed involvement of the lower uterine segment. This may be attributed to direct invasion by cervical lesions or the spread of SMMN-FGT through the cervical mucosa.

Additionally, four patients (cases 1, 5, 6, and 10) showed cystic echoes in the myometrium. These were well-defined, round, or oval, and exhibited clear acoustic transmission, with the largest diameter exceeding 1.0 cm. Pathological examination confirmed myometrial infiltration by gastric-type mucinous lesions. Therefore, differentiating G-EAC-associated cysts from adenomyoses is crucial. G-EAC cysts are well-defined with clear acoustic transmission, whereas adenomyosis-related cysts are irregular, poorly defined, and exhibit poor acoustic transmission with a “ground-glass” appearance (28, 29). Clinicians should consider the possibility of G-EAC coexisting with adenomyosis involving cystic implantation in the uterine myometrium, as observed in case 5. The patient presented with a type IV lesion characterized by an enlarged uterus and thickened myometrium, exhibiting striated acoustic shadows and small cystic echoes, whereas the cervix appeared grossly normal. Owing to the absence of sonographic features of cervical lesions, preoperative ultrasonography only diagnosed adenomyosis, potentially leading to a missed diagnosis of cervical involvement. Therefore, if a multicystic or cystic-solid lesion in the cervix accompanied by cystic or myometrial cystic features in the lower uterine segment is encountered, G-EAC should be considered. This observation represents a novel approach for improving the diagnostic specificity of G-EAC. This underscores the importance of integrating clinical characteristics and pursuing further diagnostic strategies in gynecological practice.

Notably, G-EAC may present with a normal cervix and ECF, which can cause misdiagnosis (30). In two type IV cases, ECF was initially misdiagnosed on ultrasound but subsequently confirmed via hysteroscopy and biopsy. ECF in G-EAC may result from excessive mucin production by abnormal cervical glands, tumor-induced cervical obstruction, or drainage impairment due to atrophy. Therefore, if an ECF is detected, careful ultrasonographic examination of the cervical and uterine cavity structures is essential. In the absence of abnormalities within the uterine cavity, clinicians should consider the patient’s age and history of vaginal discharge to suspect cervical lesions. During hysteroscopic curettage, routine cervical biopsies should be performed using standardized sampling techniques to prevent missed diagnoses.

Moreover, some cases exhibited a honeycomb-like pattern on ultrasonography, with small cyst clusters surrounded by larger cysts, resembling the “cosmos sign” in MRI, which may be specific for diagnosing G-EAC and aiding its differentiation from other cervical cancers (15, 20). MRI and CT are valuable for preoperative assessment of local invasion and distant metastases (14, 31). Here, MRI/CT effectively identified vaginal, parametrial, and rectal invasion, ovarian metastases, and lymph node involvement, providing critical information for clinical decision-making.

In the early stages of G-EAC, conventional cervical cancer screening methods, including cytology and biopsy, often fail due to the lesion’s deep location in the cervical canal and its mild cytological atypia. Therefore, medical imaging plays a pivotal role in diagnosis and differential assessment. CT, although rarely used due to its low soft tissue contrast, can detect multicystic or solid cervical masses with contrast enhancement improving lesion delineation. MRI, with superior soft tissue resolution, better reflects histological architecture and classifies G-EAC into four patterns, including “cosmos,” “diffuse growth,” “focal mass-like bulging,” and “solid and cystic”, thus helping distinguish benign from malignant lesions. Typically, G-EAC presents as a hyperintense multicystic mass on T2-weighted MRI with mild heterogeneous enhancement on contrast-enhanced T1 images, and the presence of solid components suggests malignancy and possible invasion. Ultrasonography, particularly grayscale and color Doppler, offers accessible and real-time visualization of cervical enlargement and multicystic lesions, sometimes mixed with solid components, with Doppler indicating vascularity. Contrast-enhanced ultrasound further enhances diagnostic accuracy by highlighting solid components within cysts, providing quantitative parameters and identifying vascular patterns suggestive of malignancy. Three-dimensional ultrasound, including 3D RealisticVue and energy Doppler, complements diagnosis by visualizing lesion morphology, blood flow distribution, and vascularization indices. Biplane transrectal ultrasound improves assessment of vaginal invasion due to its high resolution. However, ultrasound features of G-EAC may resemble benign multicystic cervical lesions, necessitating multimodal imaging and clinical correlation for accurate differentiation (14).

In addition, novel imaging modalities such as positron emission tomography-computed tomography (PET/CT) are also playing an increasingly pivotal role in the diagnosis of gastric-type endocervical adenocarcinoma (G-EAC) and in evaluating the effectiveness of chemoradiotherapy regimens (12, 32–34). PET/CT, enables noninvasive functional imaging at the molecular level. In the context of cervical cancer, FDG PET/CT has become indispensable for initial staging, restaging, and monitoring therapeutic response. The integration of PET and CT allows for the simultaneous acquisition of metabolic and anatomical data, offering superior diagnostic precision compared to CT alone—particularly in detecting metastatic involvement of regional lymph nodes and identifying extrapelvic disease spread. Reflecting its clinical significance, PET/CT has been incorporated into the National Comprehensive Cancer Network (NCCN) guidelines for staging cervical cancer from stage II to IV (32). Moreover, it is routinely employed in radiotherapy planning across multiple cancer types due to its enhanced sensitivity in identifying regions of occult tumor dissemination. Emerging evidence also suggests that PET-guided radiochemotherapy planning may contribute to improved tumor control by enabling targeted dose escalation to metabolically active tumor sites (35).

This study included young patients with G-EAC and PJS. PJS is an autosomal dominant hereditary disorder characterized by gastrointestinal polyps and pigmented macules on the skin and mucosa (4). PJS is associated with an increased risk of various malignancies, including colorectal cancer, breast cancer, and gynecologic tumors (4, 36). This case highlights the need for increased vigilance regarding the risks of G-EAC use in patients with a family history of PJS. Notably, PJS and G-EAC frequently coexisted in the same individual. Therefore, if either PJS or G-EAC is diagnosed or suspected, a thorough evaluation of the other condition is warranted (37–39).

G-EAC should be differentiated from other benign cervical lesions, such as deep Nabothian cysts, tunnel clusters, and cervical endometrial glandular hyperplasia. These benign lesions typically exhibit well-defined margins, poor vascularity, and predominantly cystic compositions with minimal or absent solid components. Moreover, they lack the characteristic imaging features of G-EAC, such as the “cosmos sign” and the “vesicular implantation sign.” CEUS may further aid in the differentiation by identifying solid components within cystic-solid lesions; in malignant lesions, the solid components tend to enhance earlier and more intensely than the surrounding normal cervical stroma, a key finding in distinguishing between benign and malignant tumors as demonstrated in our previous study. In contrast, other malignant cervical tumors often present as ill-defined solid masses with abundant intralesional blood flow and rarely exhibit multicystic or multiple cystic-solid patterns that are more specific to G-EAC (14, 15).

The diagnosis and staging of G-EAC were based on the international consensus of the 2018 International Federation of Gynecology and Obstetrics (FIGO) staging criteria (12, 13). A definitive diagnosis of G-EAC relies on histopathological examination, which may include preoperative deep cervical biopsy, cervical smear specimens, or pathological evaluation of surgically resected cervical or uterine specimens. However, previous studies have shown that preoperative pathological diagnosis identifies fewer than half of all G-EAC cases (40–43). Therefore, medical imaging methods such as ultrasound, MRI, and PET/CT have become increasingly important in the preoperative diagnosis and staging of G-EAC (12, 13). The ultrasonographic features of GEAC have demonstrated a notable correlation with pathological findings. Specifically, the presence of multiple cystic or mixed cystic-solid lesions in the cervix, particularly when the cystic components exhibit the “cosmos sign,” may serve as a relatively specific sonographic indicator of GEAC. Moreover, when cervical lesions are accompanied by vesicular implantation within the myometrium, GEAC is strongly suspected. In these cases, the clinical stage of the tumor is typically at least FIGO stage II or higher.

There is currently no universally established standard treatment for G-EAC. Management should follow the general cervical cancer treatment guidelines using individualized approaches based on pathological staging. Radical surgery is the primary treatment for patients with early stage G-EAC, followed by adjuvant chemoradiotherapy and targeted therapy. Concurrent chemoradiotherapy combined with targeted therapy is the mainstay treatment for patients with advanced-stage disease. Postoperative adjuvant therapy should be guided by the presence of intermediate- or high-risk factors, with a tendency toward broader indications owing to the aggressive nature of G-EAC. In cases where G-EAC is incidentally discovered during surgery for a presumed pelvic mass, even if the cervical lesion appears to be at an early stage, the possibility of ovarian metastasis should be carefully considered. For patients with confirmed ovarian or other resectable pelvic or abdominal metastases, cytoreductive surgery aimed at preventing gross residual disease is recommended. Chemotherapy should follow ovarian cancer regimens, which are typically a combination of paclitaxel and platinum-based agents (12, 13).

This study has several limitations. First, the small sample size of only ten patients may introduce selection bias and limit the generalizability of our findings. Second, the retrospective design of this study may have also introduced a bias. Given the rarity of G-EAC, larger multicenter studies are needed to validate the observed ultrasound features, including the “vesicular implantation sign” and the “cosmos sign,” and to confirm their diagnostic specificity and sensitivity. Prospective, large-scale multicenter studies are warranted to clarify these sonographic signs and their clinical applicability.

Overall, G-EAC predominantly occurs in postmenopausal women and is typically HPV negative. Ultrasonographic features include multicystic or cystic-solid masses in the cervix, with some lesions exhibiting vesicular implantation in the lower uterine cavity and myometrium (vesicular implantation sign). Certain cervical cystic or cystic-solid masses may present with ultrasonography characteristics resembling the “cosmos sign” in MRI, which could have diagnostic specificity. MRI and CT are valuable tools for assessing local tumor invasion and distant metastasis. Patients with a family history of PJS should be closely examined for the risk of G-EAC. The diagnosis of G-EAC requires clinical presentation, imaging findings, and pathological confirmation. Early diagnosis and treatment are crucial to improve patient outcomes.

## 5 Conclusion

Ultrasonography of G-EAC often reveals honeycomb-like clustering of small cysts surrounded by larger cystic masses in cervix (cosmos sign) and multicystic or cystic-solid lesions of the cervix accompanied by vesicle-like structures in the lower uterine cavity and/or myometrium (vesicular implantation sign), which may be specific for early diagnosis. Some G-EAC cases present with ECF as the initial symptom, which requires attention. Ultrasonography combined with hysteroscopy and biopsy can aid in early diagnosis and prevent misdiagnosis.

## Data Availability

The original contributions presented in the study are included in the article/supplementary material. Further inquiries can be directed to the corresponding authors.
